# Protective and detoxifying effects conferred by selenium against mycotoxins and livestock viruses: A review

**DOI:** 10.3389/fvets.2022.956814

**Published:** 2022-08-02

**Authors:** Manxin Fang, Wei Hu, Ben Liu

**Affiliations:** ^1^College of Life Science and Resources and Environment, Yichun University, Yichun, China; ^2^Engineering Technology Research Center of Jiangxi Universities and Colleges for Selenium Agriculture, Yichun University, Yichun, China

**Keywords:** selenium, mycotoxins, toxicity, livestock viruses, synergistic effects

## Abstract

Animal feed can easily be infected with molds during production and storage processes, and this can lead to the production of secondary metabolites, such as mycotoxins, which eventually threaten human and animal health. Furthermore, livestock production is also not free from viral infections. Under these conditions, the essential trace element, selenium (Se), can confer various biological benefits to humans and animals, especially due to its anticancer, antiviral, and antioxidant properties, as well as its ability to regulate immune responses. This article reviews the latest literature on the antagonistic effects of Se on mycotoxin toxicity and viral infections in animals. We outlined the systemic toxicity of mycotoxins and the primary mechanisms of mycotoxin-induced toxicity in this analysis. In addition, we pay close attention to how mycotoxins and viral infections in livestock interact. The use of Se supplementation against mycotoxin-induced toxicity and cattle viral infection was the topic of our final discussion. The coronavirus disease 2019 (COVID-19) pandemic, which is currently causing a health catastrophe, has altered our perspective on health concerns to one that is more holistic and increasingly embraces the One Health Concept, which acknowledges the interdependence of humans, animals, and the environment. In light of this, we have made an effort to present a thorough and wide-ranging background on the protective functions of selenium in successfully reducing mycotoxin toxicity and livestock viral infection. It concluded that mycotoxins could be systemically harmful and pose a severe risk to human and animal health. On the contrary, animal mycotoxins and viral illnesses have a close connection. Last but not least, these findings show that the interaction between Se status and host response to mycotoxins and cattle virus infection is crucial.

## Introduction

Viral infections and mycotoxin-contaminated feed are two significant issues, which are frequently encountered in the breeding industry. Indeed, while livestock and poultry are particularly vulnerable to infectious diseases caused by viruses, their feed may also not be free from mycotoxins which tend to be widely found in food grains worldwide. These fungal secondary metabolites, which include ochratoxin A (OTA), deoxynivalenol (DON), fumonisin B (FBs), T-2 toxin, zearalenone (ZEA, ZEN), and aflatoxin B1 (AFB1), can contaminate grains anywhere between their growing period and their processing, with exposure to the mycotoxins resulting in acute and chronic poisoning in both animals and humans. In this context, studies have shown that selenium (Se), a microelement first discovered in 1817 (1), is effective at reducing the toxicity of mycotoxins (2–4). This element can exist as part of organic and inorganic compounds, with Se-methylselenocysteine (MSC), seleno-L-methionine (SLM), and selenocysteine (5) representing the main organic types while selenades (e.g., H_2_Se, HSe^−^), in contrast, selenates (e.g., ${\text{SeO}}_{4}^{2-}$, ${\text{HSeO}}_{4}^{-}$, H_2_SeO_4_) and selenites (e.g., ${\text{SeO}}_{3}^{2-}$, ${\text{HSeO}}_{3}^{-}$, H_2_SeO_3_) represent major inorganic ones (6). In the year 1847, organic selenoproteins were found. A significant dietary source of selenium is selenomethionine. Plants create selenomethionine from inorganic selenium. By way of the transsulfuration route, selenomethionine is transformed in mammals into selenocysteine. By the use of selenophosphate synthase, selenocysteine is further converted into selenophosphate. It has been established that selenium plays a unique biochemical role in glutathione peroxidase (GPx), a mammalian antioxidant enzyme (7). A thorough explanation of the ping-pong mechanism of GPx-mediated peroxidase action is provided (8). Selenocysteine undergoes redox reactions in the selenolate (GPx-Se-) form. To lower the peroxide (ROOH), selenolate (GPx-Se-) is oxidized to selenenic acid (GPxSeOH). The enzyme is renewed utilizing two glutathione molecules (GSH). The GSH releases a water molecule, which then reacts to generate selenenylsulfide (GPx-Se-SG). In the following phase, GSH regenerates selenolate by forming GSSG (8). Se's antioxidant powers are the most important of all the jobs it performs. Se has been generally documented to be a powerful antioxidant against mycotoxins. Selenium protects against mycotoxin toxicity by reducing oxidative damage, decreasing pro-apoptotic proteins, and boosting immunological function in mycotoxin-affected animals. In addition, Se was also experimentally and clinically shown to be effective against viral diseases. For instance, the study by Moghaddam et al. found that 65% of the patients who succumbed to COVID-19 had low serum levels of Se, while among those who survived, only 39% had similarly low levels (9). Furthermore, Rayman et al. found that regional Se status was linked to the cure rate of COVID-19 in China (10). In summarizing the effects of Se against mycotoxin toxicity and on immune responses to viral infections, this review could be helpful to inform future studies that seek to investigate the impact of Se further.

## Systemic toxicity of mycotoxins

So far, various studies have shown that, once ingested, the mycotoxin-contaminated feed can induce adverse effects, such as hepatorenal toxicity, reproductive toxicity, cardiac and neurotoxicity, as well as immunotoxicity in animals. Further details regarding the organ systems affected by these harmful effects will now be presented in this review.

### Hepatorenal toxicity

As a major xenobiotic-metabolizing and xenobiotic detoxifying organ in the body, the liver is mainly affected after the ingestion of mycotoxins. Different studies have shown that mycotoxins can lead to hepatotoxicity. Ingesting AFB1 leads to liver poisoning characterized by necrosis, vacuolar degeneration, and bile duct hyperplasia (11), while the liver morphology of pigs was reported to be affected by low doses of DON and ZEA even after a short-term exposure (12). Further studies of the gene expression profiles of pig liver showed that DON and ZEN induced significant changes in the overall transcriptome (13). Furthermore, sows fed with diets containing DON, ZEN, and fusaric acid significantly increased hepatocyte necrosis, apoptosis, and sinusoidal leukocytes with mixed inflammatory infiltration in hepatic lobules, with apoptotic hepatocytes increasing significantly in the hepatic sinuses of experimental sows (14).

Second to the liver, the kidney is the most vulnerable organ due to its ability to filter large amount of blood and contribution to the body's homeostasis by eliminating metabolic waste products. In PK15 cells, using the increased expression of *DNMT1*, OTA can induce nephrotoxicity (15), with the underlying mechanism involving apoptosis, cell cycle arrest, DNA damage, and inhibited protein synthesis (16). The transcriptomic analysis found that hypoxia, epithelial-to-mesenchymal transition (EMT), apoptosis, and the xenobiotic metabolism pathway are affected by OTA. This study provided an opinion against OTA-induced renal toxicity through the understanding of OTA-induced EMT and apoptosis-related molecular pathway (17). OTA could induce apoptosis of HK-2 cells by enhancement of ERK 1/2 phosphorylation by activating the NF-kB pathway (18). Fumonisin B1 exposure induces apoptosis of human kidney tubular epithelial cells by regulating PTEN/PI3K/AKT signaling pathway *via* disrupting lipid raft formation (19). OTA induced apoptosis through selective endoplasmic reticulum (ER) stress activation in HK-2 cells (20). In mice, PINK1/Parkin-mediated mitophagy protects against AFB1-induced kidney injury (21).

### Enterotoxicity

Mycotoxins target the gastrointestinal tract to produce adverse effects. Ren and colleagues examined the effects of mycotoxins on the intestinal mucosal barrier and their mechanism of action. Mycotoxins were found to be capable of disrupting the intestinal mucosa's mechanical barrier function by damaging the intestinal epithelium's morphology and tissue integrity. Second, mycotoxins can alter mucin monosaccharides' composition and intestinal mucin expression, affecting mucin function. Third, the gut mucosal immune barrier function may be harmed by mycotoxins. Lastly, animals' microbiotas and ingested mycotoxins have a close relationship. The most recent research enhances the body of data connecting mycotoxins to enterotoxicity (22). For instance, through the activation of the IRE1/XBP1 pathway, T-2 toxins can destroy intestinal mucin and trigger intestinal inflammation by increased expression of inflammatory cytokines (23). Moreover, even low doses of as little as 1.0 and 5.0 mg/kg of ZEN were reported to damage the intestinal morphology of rats, to reduce the expression of mucin and the tight junction protein, to increase intestinal permeability, and to activate the RhoA/ROCK pathway (24). For the mycotoxin DON, improved NF-κB expression, disrupted jejunal mucin-2 gene expression, and reduced mucosal thickness, immune responses, and antibody titer of Newcastle disease are some of its reported effects (25). Similarly, fumonisins (FUMs) can increase NF-κB and TLR4 expression levels in the duodenum and, along with DON, can induce inflammatory reactions within that part of the body (26).

### Reproductive toxicity

AFB1 affects reproduction in different ways by causing the loss of mitochondrial functions (27) and increasing free radical production (28), with the ultimate outcomes being conditions, such as uterine diseases (29). This occurs since AFB1 can impair the growth of follicles, the survival of oocytes, and the proliferation of granulocytes while reducing the levels of gonadotropin (30). Similar observations have been made when porcine oocytes were examined, with AFB1 disrupting their maturation by disturbing mitochondrial and cytoskeletal functions, as well as cell cycle progression, to eventually induce oxidative stress and apoptosis in the oocytes (31). In the case of male rabbits, inhibited spermatogenesis and reduced reserves of testicular and epididymal sperm were noted after exposure to increasing AFB1 concentration of up to 7.5 mg/kg (32). Similarly, in sow models, ZEA could, at low doses, regulate the concentration of estrogen receptors-α and -β in specific organs while inducing apoptosis, vulva swelling, and an imbalance of reproductive hormones (33). Other mycotoxins, such as T-2, DON, and ZEN, can also cause reproductive toxicity by inhibiting steroidogenesis and cell proliferation. These were reported to lead to mitochondrial injury in porcine Leydig cells and eventually contribute to cellular apoptosis (34).

### Immunotoxicity

The AFB1 causes immunotoxic consequences, such as the disruption of innate and acquired/adaptive immunity (35–37). The Toll-like receptors (TLRs) TLR2, TLR4, and TLR7 transcription were suppressed in broilers exposed to AFB1, indicating a suppressive effect on innate immunity. These receptor proteins recognize external invaders by sentinel cells, such as macrophages and dendritic cells, as a crucial step in triggering this type of immune reaction (38). In mice-administered AFB1 dosages ranging between 5 and 75 μg/kg bw for 5 weeks, proliferation and cytokine production by splenic helper T-cells (CD4+) involved in acquired cellular immunity were likewise decreased (39), while as far as ZEA is concerned, it can induce a decrease in the IL-2 content of spleens in sows after weaning. Moreover, increased expression of IL-2β and IL-6 and reduced expression of IFN-γ have been reported (40). For instance, adding 0.1 mg/kg of ZEA to the diet of immature sows for 42 days could not only change the proportion of lymphatic T-cells and subsets of lymphatic B cells within the ileum of pigs but also change the chemical feature of gastrointestinal nerve structures (41). In 5-week-old piglets which were given a combination of ZEA and DON, the liver and lymphoid organs displayed histological changes 28 days after the treatment, with lymphocytes also undergoing significantly more apoptosis in lymph nodes and the spleen (42). Similarly, the thymus, spleen, and bursa of Fabricius of chickens which had been exposed to OTA showed pathological lesions (43). Finally, some studies assessed the adverse effects of mycotoxins using RNA-Seq data. In one such study, ZEA of different concentrations (0 mg/kg, 1 mg/kg, 2 mg/kg, and 10 mg/kg) was added to diets prior to being fed to sows which were at 8 to 14 days of gestation, and using this approach, 371 differentially expressed genes (DEGs) were identified, of which immune pathways and endometrial receptivity-related genes were the most significantly enriched (44). A similar method was applied to study AFB1's immunotoxicity to macrophages, with results indicating that AFB1 could activate various signaling pathways related to inflammatory responses. In particular, after AFB1 exposure, 25 genes related to ROS production were found and these were followed by the possible activation of seven signaling pathways linked to inflammation (45).

### Cardiotoxicity and neurotoxicity

Muthulakshmi et al. found that ZEA caused heart damage in zebrafish (46) and consuming AFB1-contaminated feed can induce perivascular cell infiltration of the heart, as well as cardiomyocyte necrosis and bleeding (47). Administering AFB1 was shown to lead to severe injuries, cardiotoxicity of cardiomyocytes, impairment of mitochondrial functions, increased production of reactive oxygen species, and apoptosis (48). Similarly, T-2 toxin and DON can significantly inhibit the mitochondrial ETC (electron transport chain) function of cardiomyocytes (49), while in zebrafish, ZEA could inhibit the activity of acetylcholinesterase (AChE), with the latter able to degrade acetylcholine to promote neuron development and nerve regeneration (46).

By crossing the blood–brain barrier, DON can cause interference with the normal functions of the nervous system (50) and the integrity of the blood–brain barrier (51), with the underlying mechanism of nerve injury likely to involve the Ca^2+^/CAM/CaMKII pathway (52). Moreover, when combined with ZEA, DON could cause brain injury in mice (53). At the same time, in the case of the T-2 toxin, its accumulation in the central nervous system (CNS) can be followed by neurotoxicity due to a similar ability to cross the blood–brain barrier (54). Some of the above effects were noted when administering different concentrations of DON (1.3 and 2.2 mg/kg) resulted in brain cell injury in weaned piglets *in vivo*.

According to previous research, the toxicity of mycotoxins in humans and livestock is a severe economic and health concern. The descriptions given above give a general idea of how dangerous mycotoxins are to human health and how they can harm animal husbandry. More research is necessary to better understand the mechanism of mycotoxin toxicity and reduce the hazards to humans and animals and the financial harm to the agricultural sector.

## Principal toxicological mechanisms induced by mycotoxins

### Oxidative stress

The imbalance between the body's antioxidant defense mechanisms and reactive oxygen species (ROS) production is known as oxidative stress. It has been suggested that it may play a role in developing various diseases. DNA damage, protein damage, and lipid damage are adverse outcomes of oxidative stress. Animals who consume FB1 experience an increase in intracellular indicators of oxidative stress, such as ROS, which creates an environment that is highly oxidized and contains more intracellular antioxidants (55). Oxidative stress develops in cells when the level of ROS exceeds the antioxidant capability. It has been postulated that FB1 causes ROS by involving the mitochondrial complex I, CYP450, and NADPH oxidase system (56). Following OTA exposure, apoptotic signal-regulated kinase 1 (ASK-1) expression is increased, which controls ROS generation and lowers mitochondrial membrane potential (57). According to the research, both *in vivo* and *in vitro*, ZEA causes DNA damage and oxidative stress (58, 59). DNA damage and elevated ROS levels were caused by ZEA and its analogs, including α-ZEL and β-ZEL (60).

### Apoptosis

Mycotoxins can cause cell apoptosis both *in vitro* and *in vivo*, a finding that more and more scientists acknowledge. OTA triggered MEK/ERK 1/2 signaling to induce apoptosis in HK-2 cells, whereas the c-MET/PI3K/AKT pathway was elevated to promote antiapoptotic survival signaling (61). Sheu et al. discovered that OTA stimulates calpain activation, which increases endoplasmic reticulum (ER) stress and apoptosis (62). A different investigation found that OTA caused nephrotoxicity and death in pig kidney epithelial cells (PK15) by activating the p38 pathway. In contrast, OTA caused immunotoxicity in porcine primary splenocytes by activating the ERK signaling system. This study made it clear that OTA caused toxicity in several cells *via* various molecular routes (63). ZEA induces apoptosis, which results in a dose- and time-dependent cell death (64). More research has shown that ZEA can lead to oxidative stress and ROS production. Overexpression contributed to the ZEA-induced cell death pathway (65, 66). Although numerous signaling pathways contribute to ZEA-induced cell death, ER stress, oxidative stress, and mitochondrial signaling pathways are the key contributors. *In vitro* results revealed that AFB1 would cause hepatocyte apoptosis *via* the death receptor pathway and mitochondrial pathway (67). HepG2 cells have significantly higher levels of expression for the transcription factors C/EBP homologous protein (CHOP), activated transcription factor 4 (ATF4), and glucose regulatory protein 78 (Bip) (68). It has been proposed that FB1's activation of the PERK-CHOP signaling pathway, which triggers apoptosis, is the cause of this (69). All of these findings demonstrated that cell apoptosis is one of the patterns of mycotoxin-induced toxicity.

### Cell cycle arrest

Many different types of research have been centered on the process of cell cycle arrest in various cells to clarify the mechanisms of mycotoxin-induced toxicity. Recent research shows that OTA causes cell cycle arrest in the G1 and G1/S phase, which is mediated by p53 and suppressed in HK-2 by cyclin D1, Cdk2, and Cdk4 (70). Duborg et al. discovered that CDK2 plays a crucial regulator in the G1 cycle arrest brought on by OTA using weight correlation network analysis. OTA inhibits expression, and CDK2 overexpression alleviates the G1 phase cycle arrest that OTA causes (71). The percentage of thymocytes and bursa of Fabricius (BF) cells in the G2M phase rose dose-dependent at 21 days of age, according to comparative effects of aflatoxin-contaminated corn on the organs in chickens. However, at 42 days old, exposure to dietary AFB1 caused cell cycle disruption in BF cells, which occurred in the G2M phase, but in the G0G1 phase in thymocytes (72). Previous studies revealed that chickens exposed to AFB-contaminated grain spleen had G2M and G0G1 phase obstruction (73). Nevertheless, several researchers demonstrated that macrophages and renal cells might experience cell cycle arrest in the S phase due to aflatoxins (74). The bursa of Fabricius of broilers subjected to dietary AFB1 revealed the underlying mechanisms of the cell cycle's G0G1 phase and G2M phase arrest. It was found that the cell cycle was stopped in the G0-G1 phase by the ATM-Chk2-cdc25-cyclin D/CDK6 pathway and the ATM-Chk2-p21-cyclin D/CDK6 route (75). AFB1 dramatically triggered S-phase arrest, which is linked to the overexpression of CDKN1A, CDKN2C, and CDKN2D, according to a different study (76). According to this research, different cell phases accumulate during AFB-induced cell cycle obstruction, which may be connected to other cells or differences between *in vivo* and *in vitro* settings.

### miRNA

A class of endogenous, non-coding tiny molecules with lengths ranging from 21 to 28 nucleotides is known as microRNAs (miRNAs). The deregulation of miRNAs, which are physiological regulators, is directly linked to the emergence of illnesses. Following OTA exposure in piglets, upregulation of miR-497, miR-133a-3p, miR-423-3p, miR-34a, and miR-542-3p and downregulation of miR-421-3p, miR-490, and miR-9840-3p altered miRNA connect to different biological pathways, suggesting that these miRNAs could be a biomarker of OTA toxicity (77). In zebrafish embryos, OTA inhibits the prolactin receptor (PrLRA) and causes brain bleeding by acting on miR-731 (78). AFB1 can significantly boost the expression of miR-33a-5p in HepG2 and block the Wnt/β-catenin signaling pathway, according to several studies (79). DON can significantly increase miR-21 levels in female pigs and activate the ERK-MAPK pathway, resulting in biotoxicity. Compared to healthy liver cells, HCC cells exhibit considerably lower levels of miR-450b-3p expression, lowered levels of its target gene PGK1's mRNA expression, and substantially decreased levels of Akt phosphorylation. This encourages the growth of HCC cells, which results in the development of HCC tumors (80). Pig *in vivo* research has demonstrated that ZEA activates the PKC and p38 signaling pathways *via* the cell membrane's GRP30 (a G-protein-coupled receptor). The FOS gene is targeted by the activated miR-7, which also detects the production and secretion of FSH and harms the reproductive health of female animals. Using an apoptotic mechanism controlled by miRNAs, ZEA can also impact reproduction (81). According to research, 30 μmol/L of ZEA *in vivo* can enhance the apoptosis gene Bad and activate caspase by activating miR-1343, miR-331-3p, and miR-744 which down-regulates the expression levels of apoptosis related genes PAK4 and ElK1 (82). These studies confirm miRNAs' significance in mycotoxins' pathophysiology and their functions as biomarkers in the prevention and regulation of toxins. To fully explain the toxicodynamics of mycotoxin, it is imperative to have a deeper insight into the role of mycotoxin-related miRNA, the interaction between miRNA and target, and the relationship between miRNA-mediated gene expression.

### Other mechanisms

One of the main mechanisms brought on by mycotoxins is the suppression of protein synthesis. The prior study examined the inhibition of protein synthesis caused by OTA in several organs, including the kidney, liver, and spleen. The ability of OTA to bind to a characteristic pocket of phenylalanine-tRNA synthase to prevent protein production was discovered by Argawal et al. using molecular docking studies (83). The FB1 causes autophagic cell death in MARC-145 monkey kidney cells, and the research of FB1-induced toxicities by altered sphingolipid metabolism found that this disruption of sphingolipid metabolism is an essential first step (84). Various downstream signaling pathways, including ER stress, PKC, and MAPKs, are activated by the disruption of sphingolipid metabolism (85), which has a wide range of biological effects, including altered cell growth and differentiation cell death, autophagy, and lipid peroxidation. It has been discovered that autophagy is crucial to numerous physiological functions. One of the most important roles of autophagy is to control cell death. Primary pig spleen lymphocytes were exposed to various DON levels. The outcomes demonstrated that DON boosted gene expression of cellular mitochondrial autophagy marker proteins LC3 and P62, a crucial indicator of autophagy induction, and produced ROS buildup in pig spleen cells (86). The most recent research suggests that many signaling pathways may be used to mediate the chronic effects of ZEA. As shown in numerous *in vivo/in vitro* settings, ZEA influences its toxicity by stimulating several signaling molecules and pathways. Using the ERs/GSK-dependent Wnt-1/β-catenin pathway, ZEA promoted ovarian follicle development in pigs (87). Recent research has shown that ZEA induces the development of neutrophil extracellular traps (NETS) by the stimulation of ERK and p38 (88). In a different study, ZEA activated the PKC, ERK, and p38 MAPK signaling pathways in pig pituitary cells to decrease the production of (follicle-stimulating hormone) FSH by GPR30 (G-protein-coupled receptor) (89). By triggering TGF-b/Smad 3 signaling in post-weaning gilts, ZEA boosted the expression of genes involved in cell proliferation and decreased the production of apoptotic proteins (90).

Both humans and animals will unavoidably consume food tainted with mycotoxins. A preventive food or feed additive, for example, could counteract mycotoxins' toxicity to safeguard both human and animal health. The discovered molecular mechanism of mycotoxins' toxicity is a significant problem that offers a variety of viewpoints and approaches. In addition, additional research is required to explain mycotoxins' precise mechanism of action despite the extensive study on their toxicity mechanisms because of the multifactorial and complex toxicology involved. At this time, further research is needed to determine how mycotoxins work together. The development of novel, sensitive analytical instruments would speed up our understanding of the toxicity processes of mycotoxins and aid in the creation of novel preventive or treatment strategies.

## The connection between viral infections in animals and mycotoxins

### Effects of mycotoxins on viral replication

Studies have been increasingly showing that viral infections and replication are influenced by various immune, nutritional, and environmental factors, including mycotoxin contamination (91, 92) and Se deficiency (93, 94). For example, 125 HIV-1-seropositive men and women drug users from Miami, Florida, were part of a study conducted over 3.5 years to determine the relationship between serum levels of Se and death, and the results indicated that the two factors were inversely related (95). Other investigations have concluded that viral infections could be the result of mycotoxins exposure and this could be observed when considering the case of porcine circovirus type 2 (PCV2), a small, non-enveloped, single-stranded circular DNA virus. Clinically, porcine circovirus-associated disease (PCVAD) is not manifested in all pigs that harbor PCV2 but studies have shown that OTA, even at low doses, could promote oxidative stress that subsequently enhances PCV2 replication *in vitro* (96–98). Since the OTA levels present in pig feed vary, this would explain, at least to some extent, why different pig farms encounter different incidence rates and severity of PCVAD (91). Further studies showed that OTA triggered ROS which, in turn, induced autophagy and promoted PCV2 replication (99). Autophagy is the main degradation system in cells, which is very important for cell survival, differentiation, and internal environment stability. DON promotes porcine epidemic diarrhea virus (PEDV) infection by triggering p38-mediated autophagy (100). Inhaled DON further affects the integrity of the respiratory epithelium of horses, thereby making them vulnerable to equine herpesvirus type 1(EHV1) infections (101). Regarding AFB1, exposure to low levels has been shown to promote infections by the swine influenza virus (SIV), as well as changes in the polarization of macrophages from M1 to M2 (102). In porcine alveolar macrophages (PAMS) and mice, AFB1 activated the TLR4-NF-κB signaling pathway that promoted SIV replication and increased associated inflammation and lung injury (103). At the same time, the T-2 toxin aggravated the pathology and pathogenesis of infectious bronchitis virus (IBV) infections (104). Moreover, in chickens, OTA and viruses responsible for inclusion body hepatitis acted synergistically to aggravate chicken anemia (105).

However, some studies have also reported that mycotoxins have no significant effects on viral infections, including inhibitory ones. For example, during *in vitro* and *in vivo* experiments, even though DON could slightly increase viral replication, it could not clearly enhance infection by the porcine circovirus (106). In addition, cells which had been infected with the porcine reproductive and respiratory syndrome virus (PRRSV) showed increased survivability after being exposed to different concentrations of DON (between 140 and 280 ng/mL). In this case, by promoting inflammation (through pro-inflammatory cytokines) and inducing apoptosis at an early stage, these concentrations effectively reduced replication of the PRRSV virus to bring about the observed effects (107). Finally, in porcine kidney (PK15) cells, the replication of the pseudorabies virus could be reduced by T-2 toxin of low concentrations (108).

### Synergistic effects between livestock viruses and mycotoxins

The synergistic effects between mycotoxins and viruses aggravate the toxic damage caused to animals. Based on previous *in vitro* and *in vivo* experiments, OTA was found to induce immunotoxicity (109, 110) while PCV2 infection could promote immunosuppression. It has also been pointed out that PCV2 infections not only worsen OTA-induced immunotoxicity but, by acting on the TLR4/NF-κB p65 signaling pathway, they could also lower the dose at which toxicity occurred (111). Such results can actually be useful to establish limits for permissible OTA levels. Other studies have found strong correlations between OTA-contaminated pig feed and porcine nephrotoxicity (112), with PCV2 infection and its resulting PDNS (porcine dermatitis and nephropathy syndrome), considered to be very common (113) and occurring through the p38-mediated autophagy (114). Increased transcription of IL-6 and IL-1β, mediated through the ERK and the p38 MAPK signal pathways, occurs due to the combined effects of DON and PCV2, although similar effects may be exerted by PCV2 through the JNK signal pathway (115). When infected with PEDV, deoxynivalenol can worsen the immunosuppression of piglets and PAMs by inhibiting the TLR4/NLRP3 signaling pathway (116). Similarly, while PRRSV infection can influence mortality, lung lesions, and weight gain, ingesting DON-contaminated diets can largely increase these effects, with additional anorectic effects also exacerbated by PRRSV infection (117).

### Mycotoxins' interference with the immunological response brought on by animal vaccines

In addition to synergistic toxicity, mycotoxins significantly affect the immune response triggered by vaccines. Indeed, providing pigs with DON-contaminated diets was shown to reduce the effectiveness of PRRSV MLV vaccines which prevent viral replication (118). Similarly, when vaccinated mice were exposed to 2.0 mg/L of DON through drinking water, the toxin disrupted their immune response by regulating the secretion of chemokines and cytokines which form part of their defensive mechanism against porcine parvovirus (119). An experimental study further showed that, in pigs, a 4-week exposure to DON reduced the efficiency of vaccines against the clinical signs of PRRSV (120). Antibody titers against porcine circovirus in the serum were also shown to decrease by 33–40% at day 28 after including 1.0 and 3.0 mg/kg of DON in diets (121). In this context, the antibody response of growing German Holstein bulls was shown to be non-linearly influenced by *Fusarium toxin* after administering vaccines against the bovine viral diarrhea virus (BVDV). Additional studies reported that strong total antibody response could be obtained when supplementing diets with 5.66 mg of DON per kg of dry matter (DM) while higher concentrations resulted in lowered total antibodies (122). Feeding diets contaminated with DON reduced the titers of serum antibodies against IBV (123), with lower concentrations of albumin (ALB) and total serum protein (TP) being some additional reported effects. Including DON in diets also reduced white blood cell (WBC) counts, lymphocyte numbers, and antibody titers against Newcastle disease virus (NDV) (26), with effects induced by AFB1 being no different as feeding diets contaminated with this toxin have been shown to decrease serum antibody titers against IBV, infectious bursal disease (IBD), and NDV (124–128). Despite the overwhelming evidence of the synergistic mechanism between mycotoxins and other microorganisms, further investigations would still be useful to further understand the process. Mycotoxin contamination of feeds and livestock virus infection have become increasingly prevalent due to the quick increase in livestock production. To determine how much mycotoxins contribute to the development of infectious diseases in animals, it is necessary to explore further the synergistic mechanism of mycotoxins against diverse pathogenic flora and bacteria.

## Underlying mechanism of Se against mycotoxins

**Figure 1:**
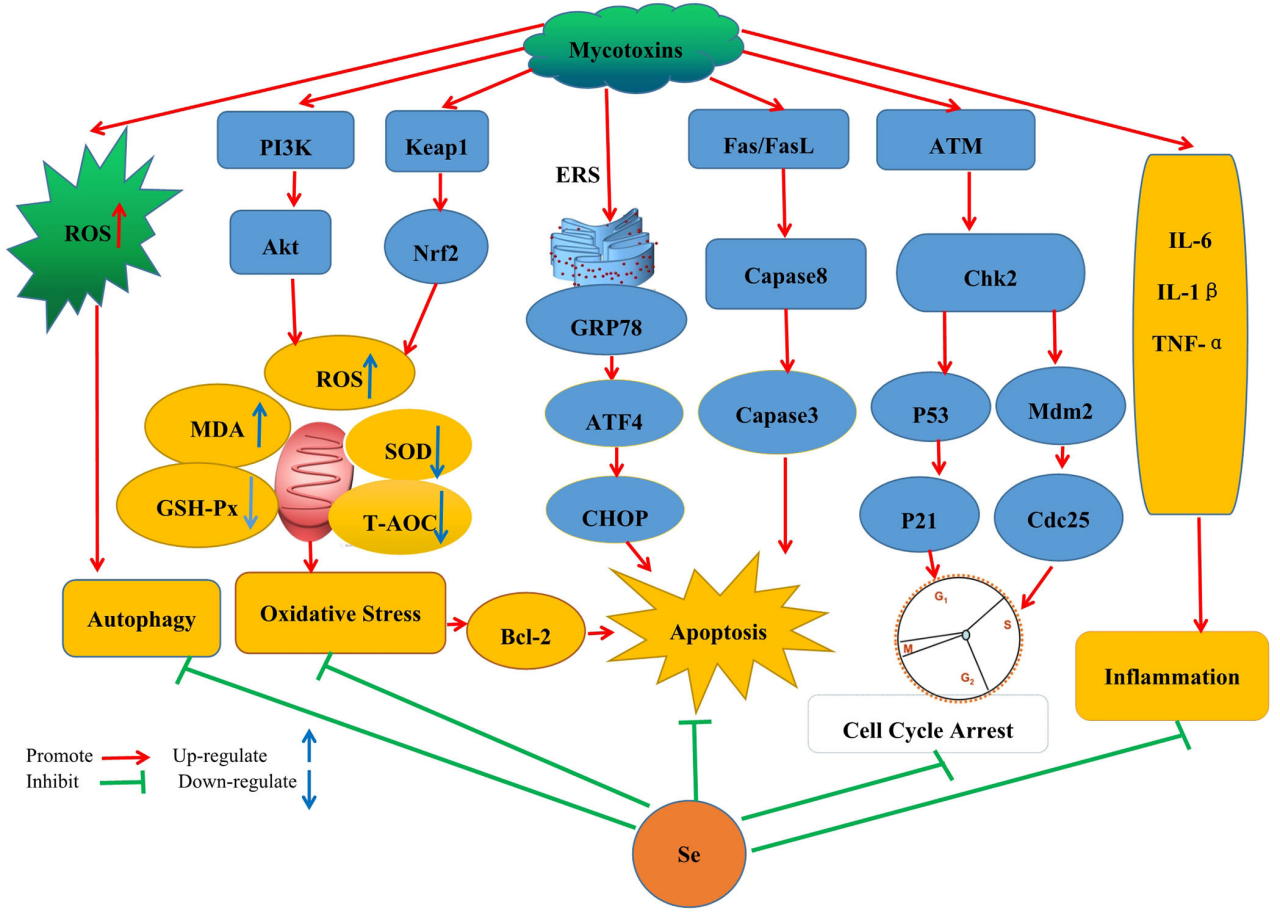
Schematic diagram of the mechanism by which SeMet improves the toxicity caused by mycotoxins by inhibiting oxidative stress, autophagy, apoptosis, cell cycle arrest, and inflammatory response pathways.

In modern breeding, harmful substances (such as mycotoxins) may be easily but inadvertently ingested, prompting the need to devise new means that would quickly help to alleviate the effects of toxins. So far, much research has been undertaken in this context, especially for developing nutritional feed supplements that would be effective for treating mycotoxin contamination without compromising nutrient loss. One example of this approach is the application of Se which is being increasingly recognized as important to reduce mycotoxin toxicity in different animal species. This review will now cover Se's specific applications and protective mechanisms in the context of mycotoxin treatments (Figure 1).

### Anti-inflammatory and anti-oxidative stress

An inflammatory response is closely related to animal exposure to mycotoxins (129–132). The application of a low dose (0.2 mg/kg) of SeMet was reported to be effective at reversing kidney injuries induced by T-2 in rabbits, with the attenuation of pathological damage, inflammatory response, and oxidative stress being some of the additional benefits of Se (133). Similarly, an Se-based compound, selenomethionine, exhibited inhibitory effects against inflammatory factors and T-2-induced oxidative stress (134). In the same vein, Se-enriched yeast (SY) was reported to antagonize OTA-induced injury to intestinal tight junctions by inhibiting the TLR4/MYD88 pathway and by extension NF-κB signaling which is a crucial mediator of inflammatory responses. In fact, within the caecum, the levels of the cytokines IFN-γ, IL-10, IL-6, and IL-1β were reversed after adding SY (135). In rabbits, improved anti-inflammatory and antioxidant processes were the primary mechanisms by which Se conferred protection against T-2 toxin (136). While in the case of chicks given Se-deficient diets, heightened spleen injuries were induced by AFB1, with the effects partly attributed to apoptosis, inflammation, oxidative stress, and six selenoproteins (137). These researches show that selenium controls immunological inflammation in a variety of ways. Selenium regulates antioxidant enzymes, which in turn contain the immune system. Inflammatory factors and inflammatory reactions can be produced when immune cells lack selenium or antioxidants. In addition, the nuclear transcription factor NF-κB is essential for expressing the immune system and the inflammatory response. The transcription factor NF-κB/p65, the expression of cellular inflammatory factors (IL-1β, IL-6, IL-11, and TNF-α), is regulated by Akana. In this study, OTA facilitated the NF-B signaling pathway's activation and inflammatory responses. On the contrary, the addition of SY reduced the activation of the NF-κB signaling pathway. These elements might be connected to selenium's anti-inflammatory properties.

High quantities of reactive oxygen species (ROS) cause oxidative stress due to an imbalance between intracellular oxygen production and the body's antioxidant system. Hydrogen peroxide (H_2_O_2_), the hydroxyl radical, malondialdehyde (MDA), glutathione peroxidase (GSH-Px), and superoxide dismutase (SOD), to mention a few, are the key indicators of the body's overall antioxidant capability. Se's antioxidant properties have long been a focus of study. Although ZEA raised the MDA content and the creatinine, urate, and blood urea nitrogen levels in mouse kidneys while lowering GSH-Px and T-SOD activity, Se mitigated these changes, causing the kidneys to recover from renal damage (138). Furthermore, for chickens, SY could regulate the Nrf2/Keap1 and PI3K/AKT pathways to reduce the hepatotoxicity and nephrotoxicity induced by ochratoxin A, while in the case of OTA-induced oxidative damage, normal kidney and liver conditions could be restored (139, 140). Other noteworthy effects include those of SeMet which can increase *SelS* and *GPx1* expression in splenocytes, as well as antioxidant capacity, to reduce immunotoxicity caused by AFB1, with GPX maintaining ROS balance by removing excess hydroperoxides (141). In addition, Se can reduce cardiomyocyte damage and cardiotoxicity induced by AFB1 by regulating redox status, with the process involving ferroptosis-related signaling, four selenoprotein genes namely *SPS2, SELENOK, TXNRD3*, and *GPX3* and four selenoproteins (142). Higher expression of *Gpx1* could represent a similar mechanism by which Se protects against OTA-induced nephrotoxic injury (143, 144). Finally, improved glutathione peroxidase activity could be the main way in which Se alleviates DON-induced damage to antioxidant enzymes (145). The primary selenium antioxidant enzyme in cells is GPX, and swine splenic lymphocytes express GPX1 at the highest levels. GPX1 knockdown decreased the protective properties of Na2SeO3 against DON-induced oxidative damage (146). Given this, Se may defend against mycotoxins by preventing the antioxidant system from becoming inactive and activating enzymes, including GPx, glutathione S-transferase (GST), SOD, GSH, and CAT.

### Anti apoptosis and regulation of endoplasmic reticulum stress

Low-doses of SeMet can significantly improve liver injury induced by T-2 along with other liver-associated conditions, such as apoptosis of hepatocytes and the level of oxidative stress. The low doses may also protect rabbit hepatocytes by inhibiting the mitochondrial-caspase apoptosis pathway (147). Some of the above benefits were, for example, shown to be possible by supplementing chickens' diets with 0.4 mg/kg of Se. In this case, the element downregulated gene expression within the death receptor pathway and the endoplasmic reticulum to alleviate, at least to some extent, the apoptosis of splenocytes induced by AFB1 (148). Improved effects of Se against AFB1-induced excessive apoptosis within the liver and the jejunum were also achieved by similar pathways (149, 150), with additional protective functions noted against liver apoptosis and hepatic dysfunctions in ducklings. Exposure to AFB1 can increase the expression levels of *p53, caspase-3*, and *Bax*, while decreasing those of *Bcl-2* and the Bcl-2/Bax ratio. Still, Se can regulate the expression of apoptosis-related proteins and reduce hepatic dysfunctions or damage in a time-dependent manner (151). In addition, groups to which Se was given had a lower percentage of bursal cells undergoing apoptosis (152). Oxidative and ER stresses largely contribute to ZEN-induced apoptosis, with Se shown to significantly prevent the same in chicken spleen lymphocytes by improving the ER stress signaling pathway (153). Similarly, when mouse kidneys were studied, Se was found to inhibit endoplasmic reticulum stress to protect against apoptosis and oxidative stress caused by ZEA (138). Indirect evidence of the protective effects of Se also comes from the fact that a deficiency of the element worsens cardiomyocyte injuries induced by T-2 by triggering more aggressive forms of endoplasmic reticulum stress. Finally, Se was reported as further promoting the expression levels of p-eIF2α, CHOP, and GRP78 (154). In conclusion, oxidative and ER stress are critical factors in mycotoxins' ability to cause apoptosis. Se dramatically reduced the ER stress signaling route and the mitochondrial-caspase apoptosis pathway, which were involved in mycotoxins' induction of apoptosis. Beyond that, Se could prevent mycotoxins from causing apoptosis by controlling the death receptors pathway at the molecular level.

### Regulation of cell cycle and autophagy

Sodium selenite, when provided as part of a diet, could effectively inhibit AFB1-induced apoptosis and block the cell cycle in the renal cells of the broiler (74). By supplementing diets with 0.4 mg/kg of sodium selenite, the jejunum of broilers could be protected from the G2/M arrest induced by 0.6 mg/kg of AFB1, with molecular regulation basically going through the ATM-Chk2-p53 and ATM-Chk2-Cdc25-cyclin B/Cdc2 pathways (155). The above dose of Se supplement was also found to reduce AFB1-induced immune toxicity in chicken's BF by alleviating oxidative damage and cell cycle arrest through an ATM-Chk2-cdc25 route and the ATM-Chk2-p21 pathway (156). According to these studies, DNA impairment plays a crucial role in the mycotoxins' ability to cause cell cycle arrest, the main target of oxidative damage in DNA. The central kinase that is activated in response to DNA damage is the ataxia telangiectasia-mutated (ATM) kinase. By controlling the ATM pathway at the molecular level, Se encouraged cell cycle recovery from the mycotoxin-induced cell cycle halt.

Autophagy occurs before apoptosis to prevent cell death. However, when unregulated, it can cause cell death, restoring the autophagy balance could help protect cells from damage. In a spermatogonia cell line, ZEA was found to induce autophagy and apoptosis (157), while in chicken granulosa cells, similar effects could occur through the MAPK and PI3K-AKT-mTOR signaling pathways (158). Similarly, OTA treatment can trigger autophagy in a dose-dependent manner (159) along with higher levels of intracellular ROS but these effects were reversed with a combination of zinc and SeMet (160). Damage caused to primary cardiomyocytes by 0.25–1 μM of T-2 toxins was shown to trigger autophagy, and since lower autophagy levels promoted T-2-induced cytotoxicity, it was considered that autophagy could protect primary cardiomyocytes from the cytotoxicity of T-2. Furthermore, in this case, the fact that Se deficiency lowered cytoprotective autophagy in the primary cardiomyocytes treated by T-2 highlights the importance of this element (161). In PK15 cells, while PCV2 replication appeared to be significantly induced by 0.1 μM of OTA, SeMet, at concentrations of 2, 4, or 6 μM, inhibited the viral replication process. OTA treatment induced autophagy by suppressing the AKT/mTOR signaling pathway, with SeMet acting through the same pathway to attenuate the effects (162). When considered as a whole, autophagy can be advantageous or harmful. In the specific study, SeMet reduced OTA-induced fibrosis by preventing ROS-dependent autophagy since exposure to mycotoxins can always cause oxidative damage that results in irregular cellular autophagy. A lack of selenium also worsens T-2 toxin-induced damage and reduces preventive autophagy. These investigations suggest that selenium has a 2-fold effect on mycotoxin-induced autophagy.

### Regulating gene expression

Gene expression is regulated by a multi-level regulatory system, with Se acting as one of the important regulatory factors. Dietary Se protected chicks from liver injury induced by AFB1 by simultaneously preventing the CYP450 isozyme-mediated conversion of AFB1 to toxic AFBO from being activated while upregulating the expression of selenoprotein genes which improve antioxidant potential through antioxidant proteins (163). In male mice, high levels of Se can also exert protective effects against reproductive damage induced by ZEN, with the effects mediated by higher expression of the *Vim* and *Cdh2* genes (164). Similarly, by stimulating the expression of synthetic testosterone enzymes and steroidogenic acute regulatory protein (StAR) to improve antioxidant and testosterone levels, Se can protect against testicular toxicity induced by AFB1 (165). Female mice were not excluded from the potential benefits of Se as the element could improve conditions, such as necrosis of human endometrial microvascular endothelial cells (HEMECs) and uterine injury which had been induced by AFB1. For instance, a significant increase in the expression of *MLKL, RIPK3*, and *RIPK1* was observed in HEMECs after exposure to 10 μM of AFB1 and subsequent improvement occurred after treatment with 5 μM of Se (166). Furthermore, T-2-induced blood abnormalities were also improved by SeMet, with damage to the spleen and thymus tissues reduced along with a significant increase in the expression of proliferating cell nuclear antigen (PCNA) within tissues (136). Finally, SY could reverse any injury caused to the intestinal barrier after exposure to OTA by acting on the TLR4/MYD88 pathway to prevent NF-κB expression while increasing that of the tight junction-related genes *ZO-1, Occludin*, and *Claudin-1* (135). These investigations demonstrated that the expression levels of the selenoprotein mRNA transcripts could be determined by selenium status. The selenium level influences non-selenoprotein gene expression as well. It is necessary to conduct more studies to comprehend the interconnected pathways and activities better.

### Enhanced immune function

Recent research on selenium's antagonistic effects against mycotoxins shows that the element can enhance immune functions. As previously pointed out, diets containing as low as 0.3 mg/kg of AFB1 could increase apoptosis of thymocytes, induce histopathological lesions of the thymus, and lower the number of mature thymocytes. However, supplying sodium selenite as part of the diet can reverse the above effects (167). In particular, supplementing diets with 0.4 mg/kg of Se could protect against impaired humoral or mucosal immune functions caused by AFB1. In other cases, such supplementation increased IgA+ cell numbers and the IgM, IgG, IgA, and sIgA content (168). Furthermore, an improved immune function could be achieved with sodium selenite which was found to not only reduce histopathological damages to the spleen but also increase the latter's relative weight and increase in the number of splenic T-cell subsets (169). Changes in the number of T-cell subsets, the CD4+/CD8+ ratio, and cytokine expression were also some additional effects reported by Se supplementation after AFB1 treatment, with the changes restoring the above features close to those of a control group without exhibiting toxicity to the cecal tonsil (170). In chickens, 0.6 and 0.8 mg/kg of Se could improve immune function by increasing the IFN-c and IL-2 serum levels along with more significant numbers of peripheral blood T-cell subsets (171). As far as DON was concerned, even though it could induce a significant dose-dependent decrease in the levels of proteins, mRNA, IgM, IgG, IFN-γ, IL-10, IL-6, IL-4, and IL-2, its simultaneous administration with Se resulted in a significant increase in the levels of all factors studied, including proteins and mRNA. Hence, selenium's potential to counter immunosuppression and other associated negative outcomes induced by DON makes it a promising candidate to be considered against DON-mediated toxicity (172). Furthermore, Se can further confer protection against the immunotoxicity of T-2 (173). For instance, although administering T-2 at a sublethal concentration resulted in lower numbers of B cells (CD19^+^), opposite effects were noted when Se was injected simultaneously or 24 h prior to the toxin. Since the results pointed out the ability of selenium to inhibit a reduction in B lymphocytes (CD19^+^), it was an indication of its suitability to alter B-lymphocyte subsets after exposure to T-2 (174). To summarize, selenium has a more substantial immunomodulatory effect on the immune function changes caused by mycotoxins. The antioxidant activity of Se plays an essential role in boosting immunological function. Proteins and nucleic acids can be damaged by oxidative stress caused by high quantities of reactive oxygen species. Se may work by decreasing reactive oxygen species generation and mitochondrial dysfunction induced by mycotoxins, enhancing cell viability and function, and antagonizing mycotoxins' suppressive effects on immunological responses.

Among the many challenges facing the breeding industry, exposure to mycotoxins represents a significant concern in livestock and poultry production. Nutritional supplementation could be an efficient and cost-effective approach to reducing the widely known toxic effects of AFB1. As a potential antioxidant, Se is involved in different parts of biological systems, representing an important component of several essential enzymes. As such, various studies are being undertaken globally to exploit its benefits against mycotoxins better. This could potentially be useful in future to improve both human and animal health while avoiding economic losses worldwide.

## Role of Se in livestock virus infection and enhancing immune response

**Figure 2:**
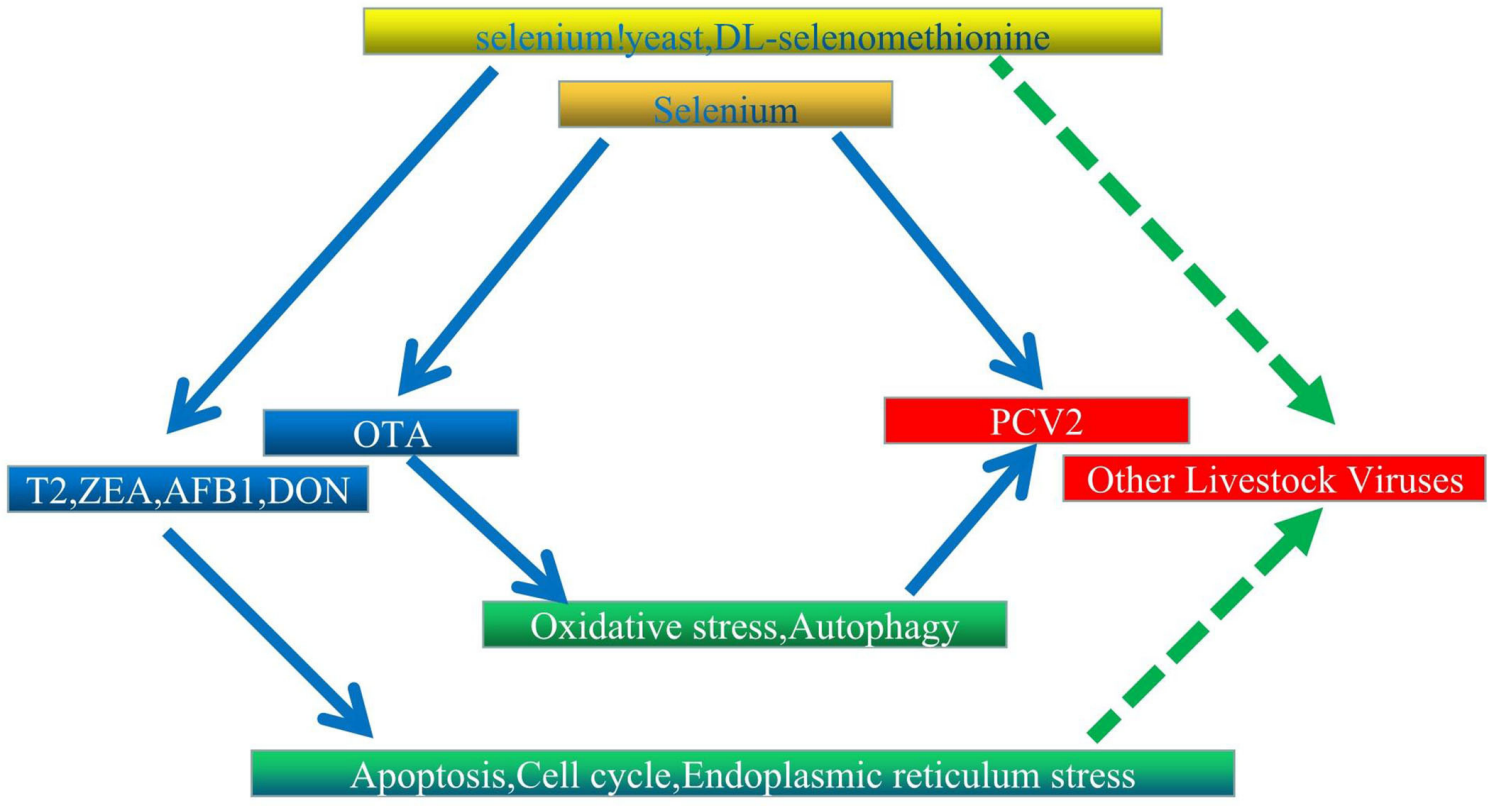
Proposed interaction network between Se, mycotoxins, and livestock virus. Solid and dashed arrows represent verified direct and potentially indirect connections, respectively.

The Se is historically known to lower the incidence and severity of viral infections (175–179). However, its deficiency may alter immune functions (180), the expression of relevant selenoproteins (181), the virulence of viruses (182), and antioxidant responses (183), thereby causing humans and animals to be more susceptible to severe viral and bacterial infections (184). In addition, experimental and clinical data confirm that this element can be effective against viruses, such as Ebola and hantavirus (185, 186). In livestock, DL-selenomethionine can enhance the activity of glutathione peroxidase by inhibiting PCV2's processes in a concentration-dependent manner (187). Similarly, Se could increase *GPx1* expression to prevent PCV2 from replicating as a result of oxidative stress (35). At the same time, systemic inflammation was altered in the case of Se yeast, and typical organ morphologies were maintained to reduce PCV2 infection (188). Selenoproteins S (sels) can also mediate protective functions through their antioxidant potential, and this was observed when its overexpression in pigs inhibited p38 phosphorylation in PK15 cells and oxidative stress to block OTA-induced PCV2 replication (189). However, in this case, activation of the AKT/mTOR signaling pathway to inhibit autophagy is also recognized as an alternative protective mechanism (162). In a different type of mechanism, selenizing astragalus polysaccharide can activate the PI3K/AKT pathway to inhibit autophagy, thereby preventing PCV2 from replicating as a result of oxidative stress (190). In other investigations, SeMet at doses higher than physiological (16 μM) inhibited the replication of porcine delta coronavirus in pig kidney epithelial (LLC-PK) cells (191). Finally, supplementing diets with Se was found to reduce virus shedding and improved *ISG* and *IFN* expression. Since supplementation of chicken diets with Se can also enhance defensive mechanisms against viruses, this could be exploited for controlling viral infections in poultry (192). According to the findings, OTA therapy causes oxidative stress and enhances PCV2 replication. Selenium inhibits oxidative stress-induced promotion of PCV2 replication by improving GPx1 expression, and overexpression of pig selenoprotein S inhibits OTA-induced promotion of PCV2 reproduction by inhibiting oxidative stress and p38 phosphorylation; these findings may serve as a foundation for further research into the biological function of selenium and the control of livestock virus infection. We believe that further research into the association between various mycotoxins and viruses and the protective mechanism of selenium is required (Figure 2).

In addition to inhibiting viral infections, Se also enhances the immune response of vaccines. In this context, higher mice resistance was noted after immunization with a PrV vaccine when Ginseng Stem-and-Leaf Saponin (GSLS) and Se were combined (193). Furthermore, after administering a vaccine consisting of killed Newcastle and Avian Influenza viruses, excellent antibody titers were obtained, especially when the ISA70VG adjuvant was combined with Nano-Se (194). Similarly, supplementing diets with Se, especially organic ones (SEY), can be effective against H9N2 AIV of low pathogenicity due to improved effectiveness and immunogenicity of a formalin-inactivated vaccine (195). In this context, the effects of Se supplementation are particularly evident when they are considered along with those of GSLS. For instance, GSLS-Se was not only found to promote strong antibody responses, especially NDV- and IBV-specific ones, but also to trigger the response at an early stage while prolonging its duration. Moreover, enhanced IL-4 and IFN-γ production, increased proliferation of lymphocytes, modulation of immune-related enzymes, and regulation of antioxidant mechanisms represent some of the additional effects induced by GSLS-Se (196). Finally, during the immunization of young chicks, higher iNDV-specific HI titers were observed when an NDV vaccine was administered jointly with GSLS-Se, with the latter also producing significantly more NDV-specific sIgA, as well as IgM^+^, IgA^+^, and IgG^+^ plasma cells (197).

In brief, the fact that Se confers protection to the host and alleviates adverse outcomes during viral infections justifies the potential of considering Se-based interventions in future.

## Conclusion and future perspectives

High rates of viral infections and the high toxicity of mycotoxin seriously threaten the production performance of animals and human health, prompting the need to find means for solving the issue. In this context, Se can be an effective antioxidant whose antagonistic effects against mycotoxin toxicity, virus infection, and immunity have attracted extensive attention. This review provided a comprehensive and an extensive background on the protective role of Se in effectively reducing mycotoxin toxicity and viral infections in animals. Based on the above literature, it could roughly summarize that there is a close relationship between mycotoxins and livestock viral diseases, and Se has a certain antagonistic effect on the toxicity of mycotoxins and livestock viruses infection. As both mycotoxins and infection of livestock viruses could induce oxidative damage, ER stress and autophagy, which play important roles in the viral replication, the synergistic mechanisms of livestock viruses infection with mycotoxins deserve to be further investigated. At present, while research on the role and mechanism of Se in alleviating mycotoxin toxicity is comprehensive, similar research on its role in preventing animal infections and in driving immunity is relatively limited, with even less attention paid to the mechanism, However, it is expected that the role of Se in alleviating mycotoxin toxicity, resisting animal virus infection, and enhancing vaccine immune response will gain greater importance as part of future studies. Selenium, on the contrary, is a double-edged sword. Se is an indispensable trace element with a fine line between its positive and harmful effects. The proposed research suggests that selenium is one of the most promising agents to be studied for its protective effects against the toxic effects induced by mycotoxins, with the caveat that its impact is dependent on many variables, such as its chemical form as well as the applied dose and experimental model, making the selection of the most effective supplement a very complex issue, so supplementation must be carried out with caution to obtain the best results.

## Author contributions

MF and WH conceived and designed the review. MF and BL provided manuscript editing. All authors discussed, critically revised the contents, and approved the final manuscript.

## Funding

This work was supported by the Science and Technology Project of Jiangxi Provincial Department of Education (GJJ211632) and the Initial Scientific Research Fund of Yichun University (3360119046).

## Conflict of interest

The authors declare that the research was conducted in the absence of any commercial or financial relationships that could be construed as a potential conflict of interest.

## Publisher's note

All claims expressed in this article are solely those of the authors and do not necessarily represent those of their affiliated organizations, or those of the publisher, the editors and the reviewers. Any product that may be evaluated in this article, or claim that may be made by its manufacturer, is not guaranteed or endorsed by the publisher.
